# Measurement of sulfur content in coal mining areas by using field-remote sensing data and an integrated deep learning model

**DOI:** 10.7717/peerj-cs.2458

**Published:** 2024-11-04

**Authors:** Jingyi Liu, Ba Tuan Le

**Affiliations:** 1 College of Sciences, Northeastern University, Shenyang, China; 2 Artificial Intelligence Laboratory, Control, Automation in Production and Improvement of Technology Institute, Hanoi, Vietnam

**Keywords:** Neural network, Remote sensing, Coal, Vis-NIR spectroscopy

## Abstract

High-quality coal emits a smaller amount of harmful substances during the combustion process, which greatly reduces the environmental hazard. The sulfur content of coal is one of the important indicators that determine coal quality. The world’s demand for high-quality coal is increasing. This is challenging for the coal mining industry. Therefore, how to quickly determine the sulfur content of coal in coal mining areas has always been a research difficulty. This study is the first to map the distribution of sulfur content in opencast coal mines using field-remote sensing data, and propose a novel method for evaluating coal mine composition. We collected remote sensing, field visible and near-infrared (Vis-NIR) spectroscopy data and built analytical models based on a tiny neural network based on the convolutional neural network. The experimental results show that the proposed method can effectively analyze the coal sulfur content. The coal recognition accuracy is 99.65%, the root-mean-square error is 0.073 and the R is 0.87, and is better than support vector machines and partial least squares methods. Compared with traditional methods, the proposed method shows many advantages and superior performance.

## Introduction

Coal has always been the world’s main energy source. The world’s demand for high-quality coal is increasing. High-quality coal emits a smaller amount of harmful substances during the combustion process, which greatly reduces the environmental hazard. How to quickly determine the quality of coal in coal mining areas has always been an important research issue. This is of great significance for improving mining speed, reducing costs and protecting the environment. In recent years, computers, remote sensing and spectroscopy have developed rapidly. Therefore, we need to combine these technologies to solve the problem of quickly determining coal quality.

Remote sensing plays an important role in coal exploration and monitoring (Thiruchittampalam et al., 2024; Tan & Qiao, 2020; Biswal, Raval & Gorai, 2019; Le et al., 2019). In the process of coal mining and production, real-time division and identification of coal mines have important guiding significance for production planning and resource assessment. Remote sensing can overcome the complex and steep terrain of mining areas, and it can monitor and evaluate coal mining areas quickly, low-cost, and large-scale (He et al., 2019). Zeng et al. (2017) mapped the opencast coal mining areas based on remote sensing images and machine learning algorithms, and the recognition accuracy was 97.07%. Madhuanand et al. (2021) used satellite imagery and a deep neural network to determine the surface distribution of coal mining areas with an overall accuracy of 95%. Ma et al. (2021) conducted a comprehensive assessment of the Mongolian Plateau opencast coal mine based on remote sensing and *in situ* data. Their findings show that over the past 40 years, the number of opencast coal mines in the region has increased 21-fold and the area 33-fold. Luther et al. (2022) made observations of methane emissions from Polish coal mines using ground and remote sensing data. This study shows that the methane emissions are higher than the European Pollutant Release and Transfer Register (E-PRTR). Ali et al. (2022) monitored the mining and reclamation of open pit coal mines in Pakistan based on the Landsat remote sensing data. This study facilitates the monitoring of land cover change in coal mines and demonstrates that remote sensing can be used to monitor activity in coal mining areas.

Spectroscopy has played an important role in coal composition analysis due to its low cost and rapid detection (Sheta et al., 2019; Chen, Tang & Guo, 2022; Begum et al., 2021). Song et al. (2022) performed coal gangue composition analysis using thermal infrared spectroscopy and a spatial attention network. The effectiveness of the proposed method is demonstrated by comparing multiple models. This provides a low-cost, efficient and reliable method for coal gangue analysis. Xiao et al. (2022) proposed a coal identification method based on visible-infrared spectroscopy and deep learning. They first converted the 1D spectra to 2D data. Then use convolutional neural network to extract high-level features of the data. Finally, a machine learning method is used to classify the features. The results show that their proposed method achieves 97.6% accuracy. Petrovic et al. (2022) used laser-induced breakdown spectroscopy for quantitative analysis of coal. The results help control the coal combustion process in power plants. Hou et al. (2022) rapid measurement of coking coal characteristics based on laser-induced breakdown spectroscopy. In the experiment they measured two parameters including cohesion index and maximum plastic layer thickness. The results show that their proposed method can quickly measure the two parameters of coking coal.

The sulfur content in coal includes both organic and inorganic sulfur, which is collectively called total sulfur. Sulfur is a harmful element, and sulfur dioxide emitted after coal combustion will cause air pollution and damage the ecological environment. Therefore, sulfur content is an important parameter to identify coal quality (Cai et al., 2021; Tang et al., 2020). Many studies have shown that the Vis-NIR spectroscopy can be used to quickly detect sulfur content in coal (Le et al., 2018a; Yan et al., 2017). Song et al. (2021) developed an online system for analyzing sulfur content in coal, which was evaluated in an actual power plant and obtained objective results. Sarıhan et al. (2021) studied the combustibility and non-combustibility of sulfur in coal and proposed a new method to determine it.

As for mapping coal quality using remote sensing, the low resolution and band characteristics of satellite remote sensing images increase the difficulty of analyzing coal quality. Deep learning has developed rapidly in remote sensing, but most of the research is only theoretical results (Cheng et al., 2020; Albarakati et al., 2024; Su et al., 2024). In solving practical problems, deep learning in remote sensing will face many problems such as label noise, the combination of space and ground samples, *etc*. This study will discuss the application of deep learning to a practical problem and propose a method for mapping sulfur in coal. Firstly, we acquire field Vis-NIR spectroscopy and remote sensing data. Secondly, through spatial transformation and data augmentation methods, we transform field Vis-NIR spectroscopy data into spatial-spectral data, and this data can be processed using deep learning. Thirdly, we propose a tiny deep neural network and establish a coal analysis model. Coal and non-coal areas can be classified by the model. Finally, we construct a model for mapping sulfur content in coal and verify the effectiveness of the proposed method through experiments.

## Data and methods

### Data

#### Research area

The research area is the Baorixile opencast coal mine. The mining area is high in the north and low in the south, and the coal seam structure is relatively simple. The maximum and minimum mineable thickness of the coal seam are 28 and 7 m respectively, with an average thickness of 22 m. The reserves of coal resources are about 4.2 billion tons, and the calorific value of coal is 3,700–4,300 kcal/kg. Coal has the characteristics of low ash, high volatile content, low sulfur, low phosphorus, and low harmful components.

#### Field Vis-NIR spectroscopy data

Coal and non-coal samples collected at the Baorixile opencast coal mine. Spectral data of the samples were measured using the SVC HR-1024 ground object spectrometer. The experiment was carried out outdoors, using solar radiation as the light source, the observation time was selected between 10:00–14:00 h, and the sky was clear. The coal sample surface was kept horizontal, and the spectrometer lens and the observation surface were kept vertical. Data errors caused by interference from other light sources should be minimized. Since the sun’s radiation values are different at different times, we need to calibrate the spectrometer with a whiteboard every 10 min in the experiment.

#### Remote sensing data

This research uses Landsat eight remote sensing imagery as the data source. Coal mine remote sensing data were collected in 2018 and 2021. Before using the remote sensing data, it needs to be preprocessed accordingly, mainly including radiometric calibration, atmospheric correction and image fusion (Le et al., 2019). The preprocessed remote sensing data is closer to the field Vis-NIR spectroscopy data. Finally, the field Vis-NIR spectroscopy data and remote sensing data need to be matched to ensure that the two data sources are compatible and facilitate subsequent processing.

### Basic knowledge

#### Convolutional neural network

Convolutional neural network is mainly composed of input layer, convolution layer, pooling layer and fully connected layer (Albarakati et al., 2024; Su et al., 2024). By stacking these layers together, a deep network can be constructed. The convolutional and fully connected layers perform transformation operations on the data and contain most of the parameters of the network. The pooling layer is generally a fixed function.

#### Double layer extreme learning machine

Double extreme learning machine (DELM) training process is demonstrated as follows.

(1) $${f_{ELM - L}}({\rm x}) = \sum\limits_{j = 1}^L {{\beta _j}g({{\bf w}_j} \cdot {{\bf x}_i} + {b_j}) = {{\bf t}_i}} ;\,\,{b_j},{\beta _j} \in {\bf R}$$

where ${{\bf x}_i} = {[{x_{i1}},{x_{i2}}, \cdot \cdot \cdot ,{x_{in}}]^T} \in {{\bf R}^n}$, ${{\bf t}_i} = {[{t_{i1}},{t_{i2}}, \cdot \cdot \cdot ,{t_{im}}]^T} \in {{\bf R}^m}$, ${{\bf w}_j} = {[{w_{j1}},{w_{j2}}, \cdot \cdot \cdot ,{w_{jn}}]^T}  \in {{\bf R}^n}$ is the input weight, *L* is the hidden layer nodes, $g( \bullet )$ is the activation function, ${\beta _j}$ is the output weight.

The output weight of the first layer is ${{\rm \beta }_1}$ and the output weight of the second layer is ${{\rm \beta }_2}$, the calculation method for these two matrices is derived from the study (Qu et al., 2016).

(2) $${{\rm \beta }_{\rm 1}} = {\bf H}_1^{\rm + }{\bf T}$$

(3) $${{\rm \beta }_2} = {\bf H}_2^ + {\bf T}.$$

### Proposed method

#### Data augmentation

Deep learning is sensitive to spatial-spectral information, so deep learning is highly recognized in remote sensing. Field Vis-NIR spectroscopy data is purely spectral data without spatial information, and remote sensing data contains spectral and spatial information. Therefore, we need to convert field Vis-NIR spectroscopy data into spatial-spectral data, so that field Vis-NIR spectroscopy data can be combined with remote sensing data, and deep learning algorithms can be used for data analysis (Xiao et al., 2023). This study uses a multidimensional random generation method to transform field Vis-NIR spectroscopy data into spatial-spectral data. Each field spectral data is regarded as a pixel, and a multidimensional matrix is generated according to a certain proportion of each class. This matrix satisfies the data input form required by deep learning, and solves the problem of combining field Vis-NIR spectroscopy data and remote sensing data.

#### Multilayer integrated DELM

The multilayer integrated (MI) algorithm design inspired by multi-layer neural network and random forest algorithms (Elhaki & Shojaei, 2022; Bai et al., 2022). The algorithm structure can be divided into multiple layers and multiple nodes. There are multiple branches at each node. In the MI algorithm structure, the initial layer contains multiple intelligent algorithms. We assume that the number of layers is K, and each node has m branches, then the number of nodes ${{\rm S}_i}$ of the *i*-th layer and the number of nodes S of the entire algorithm are represented by Eqs. (4) and (5), here (i = 1, 2,…, K).

(4) $${{\rm S}_i} = {m^{i{\rm - 1}}}\,$$

(5) $${\rm S} = {m^0} + {m^1} + ... + {m^{{\rm K - 1}}}.$$

We assume a classification problem with C class, ${c_t} \in (1,2,...,C)$; the true attribute for a sample ${x^{test}}$ is c, the probability of ${x^{test}} = q\,(q \in (1,2,...C))$ is $P_i^{j,m}(q|{x^{test}})$, then $P_i^{j,m}(q|{x^{test}})$ is represented by Eq. (6).

(6) $$P_i^{j,m}(q|{x^{test}}) = \displaystyle{{Su{m_{m,{x^{test}}}}(q)} \over m}.$$

Here, $Su{m_{m,{x^{test}}}}(q)$ is the classification result of ${x^{test}}$ in branch *j*-th; $j = 1,...,{{\rm S}_i}$.

If $P_{i + 1}^{j(i + 1),m}(c|{x^{test}}) \; > \; \max (P_{i + 1}^{j(i + 1),m}({c_t}|{x^{test}})),c \ne {c_t}$, then there exists an $\epsilon \; > \; 0$ such that inequality Eq. (7) holds:

(7) $$P_{i + 1}^{j (i + 1),m}(c|{x^{test}}) - P_{i + 1}^{j(i + 1),m}({c_1}|{x^{test}}) \; > \; \epsilon$$

For a sufficiently large m, we can expect Eqs. (8), (9) to hold.

(8) $$\mathop {\lim }\limits_{m \to \infty } P_i^{j,m}(q|{x^{test}}) = P_{i + 1}^{j(i + 1),m}(q|{x^{test}})$$

(9) $$\sum\limits_{q = 1,...,C} {P_i^{j,m}(q|{x^{test}})} = 1 .$$

From the definition of the limit (Cao et al., 2012), for each class of q, any given positive scalar $\displaystyle{1 \over C}\epsilon \ge \delta \; > \; 0$ can be obtained, and there is a positive integer ${m_{0q}}$, when ${m_q} \ge {m_{0q}}$ is true, inequality Eq. (10) holds.

(10) $${\rm |}P_i^{j,{m_q}}(q|{x^{test}}) - P_{i + 1}^{j(i + 1),{m_q}}(q|{x^{test}}){\rm | \; < \; }\delta.$$

For all ${m_q}$, let $m = Max\{ {m_q}\}$, then inequality Eq. (11) holds.

(11) $${\rm |}P_i^{j,m}(q|{x^{test}}) - P_{i + 1}^{j(i + 1),m}(q|{x^{test}}){\rm | }\; < \; \delta.$$

When *q = c*, inequality Eq. (12) can be obtained.

(12) $$P_{i + 1}^{j(i + 1),m}(c|{x^{test}}) - \delta \; < \; P_i^{j,m}(c|{x^{test}}) \; < \; P_{i + 1}^{j(i + 1),m}(c|{x^{test}}) + \delta.$$

For any ${c_t} \in (1,2,...,C)$ and ${c_t} \ne c$, we have Eq. (13).

(13) $$P_i^{j,m}({c_t}|{x^{test}}) = 1 - P_i^{j,m}(c|{x^{test}}) - \sum\limits_{q \ne c,{c_t}} {P_i^{j,m}(q|{x^{test}})}.$$

Equations (14) and (15) can be obtained by Eqs. (11) and (12).

**Algorithm 1:** MI-DELM algorithm.

1. Input data;
2. Initialize the MI algorithm and determine ${{\rm K}_{{\rm MI}}}$ and m;
3. Initialize multiple DELM algorithms;
4. DELM:
**for** $\rm i = 1:1:m.^{({{\rm K}_{{\rm TR}}}-1)}$
**T $\Leftarrow \text{T_i}\Leftarrow\text{DELMs}$**
**end**
5.IM:
$\rm kk = {{\rm K}_{{\rm MI}}} -1$
**while** (kk > 0)
** for** i1 = 1:1:q
** for** i2 = 1:1:p
$\text{C}\Leftarrow$ Eq. (6)
** end for**
** end for**
kk = kk − 1

(14) $$P_i^{j,m}({c_t}|{x^{test}}) \; < \; 1 - P_{i + 1}^{j(i + 1),m}(c|{x^{test}}) + \delta - \sum\limits_{q \ne c,{c_t}} {P_i^{j,m}(q|{x^{test}})}$$

(15) $$P_i^{j,m}({c_t}|{x^{test}}) \; < \; P_{i + 1}^{j(i + 1),m}({c_t}|{x^{test}}) + (C{\rm - 1)}\delta.$$

The inequality Eq. (16) can be obtained through inequalities Eqs. (7), (12) and (15):

(16) $$\eqalign{& P_i^{j,m}(c|{x^{test}}) \; > \; P_{i + 1}^{j(i + 1),m}({c_1}|{x^{test}}) + \epsilon - \delta \ge P_{i + 1}^{j(i + 1),m}({c_t}|{x^{test}}) + (C - 1)\delta \cr & \; > \; P_i^{j,m}({c_t}|{x^{test}}).}$$

It can be seen from the inequality Eq. (16) that when m is large enough, the probability that the predicted sample ${x^{test}}$ belongs to class c is the largest. Therefore, the MI algorithm adopts the method of majority voting and can make a correct prediction with a probability of 1. We combine this algorithm with DELM, and DELM serves as the initial layer of MI algorithm, called MI-DELM algorithm. The algorithm implementation process is as follows:

#### Build analytical model

Since the remote sensing images contain many different objects, the sulfur content in coal cannot be predicted directly using regression models. Therefore, we first extracted coal regions using a classification model, and then used a regression model to measure sulfur content. We propose a coal classification model based on CNN and MI-DELM algorithms. For the regression model, we built a prediction model of coal content based on the DELM algorithm. However, because the field Vis-NIR spectroscopy data is limited, the regression effect is not very good. Therefore, we propose an ensemble DELM algorithm to improve the predictive ability of the regression model. Previous studies have demonstrated that ensemble systems can effectively enhance model generalization (Le et al., 2018b). Since the weight and bias of DELM are randomly given, a set of multiple DELM models are randomly generated for training. Euclidean distance is then used to determine the weights and biases of the best models. Finally, we combine these best models into a large model. The final result is the average of the summed results of each best model.

**Figure 1:**
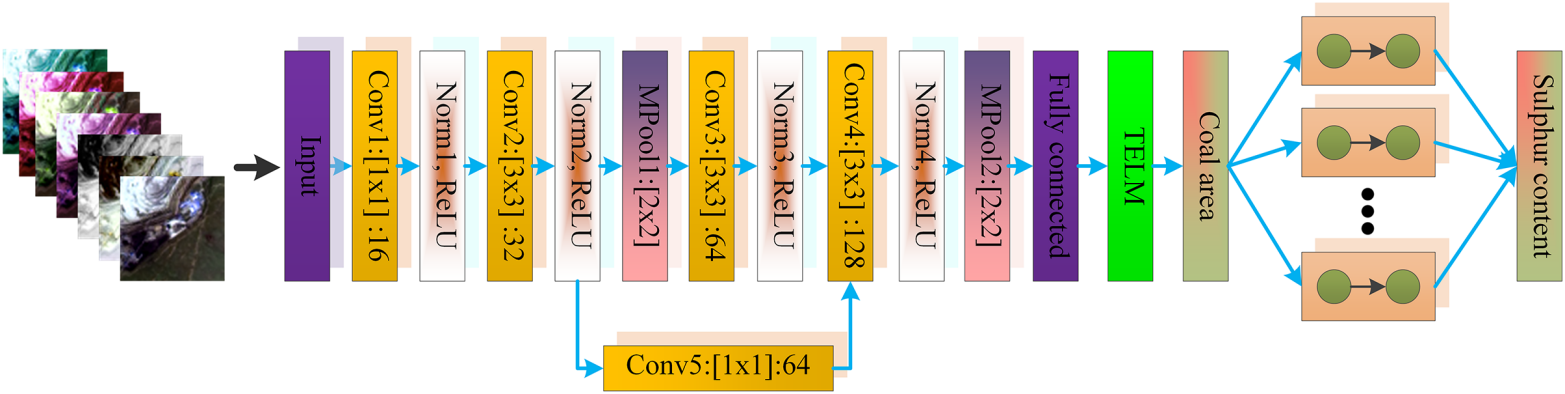
Coal sulfur content measurement model.

Figure 1 is the structure of our proposed model, including five convolutional layers, four normalization layers and two pooling layers, and ReLU function as activation function. The convolutional kernel size of the first convolutional layer is 1 × 1, and the remaining convolutional layers have a convolutional kernel size of 3 × 3. The fifth layer in the model is the residual layer, which is used to prevent problems such as model degradation and gradient disappearance. The second and fourth convolutional layers are followed by a pooling layer with a pooling kernel size of 2 × 2. After the features are extracted by each CNN layer, the output features are classified using the MI-DELM algorithm and the coal area is obtained. Finally, the ensemble DELM algorithm is used to predict the sulfur content of the coal area, and the distribution of the coal mining area is obtained.

## Results and discussion

### Classification performance

**Figure 2:**
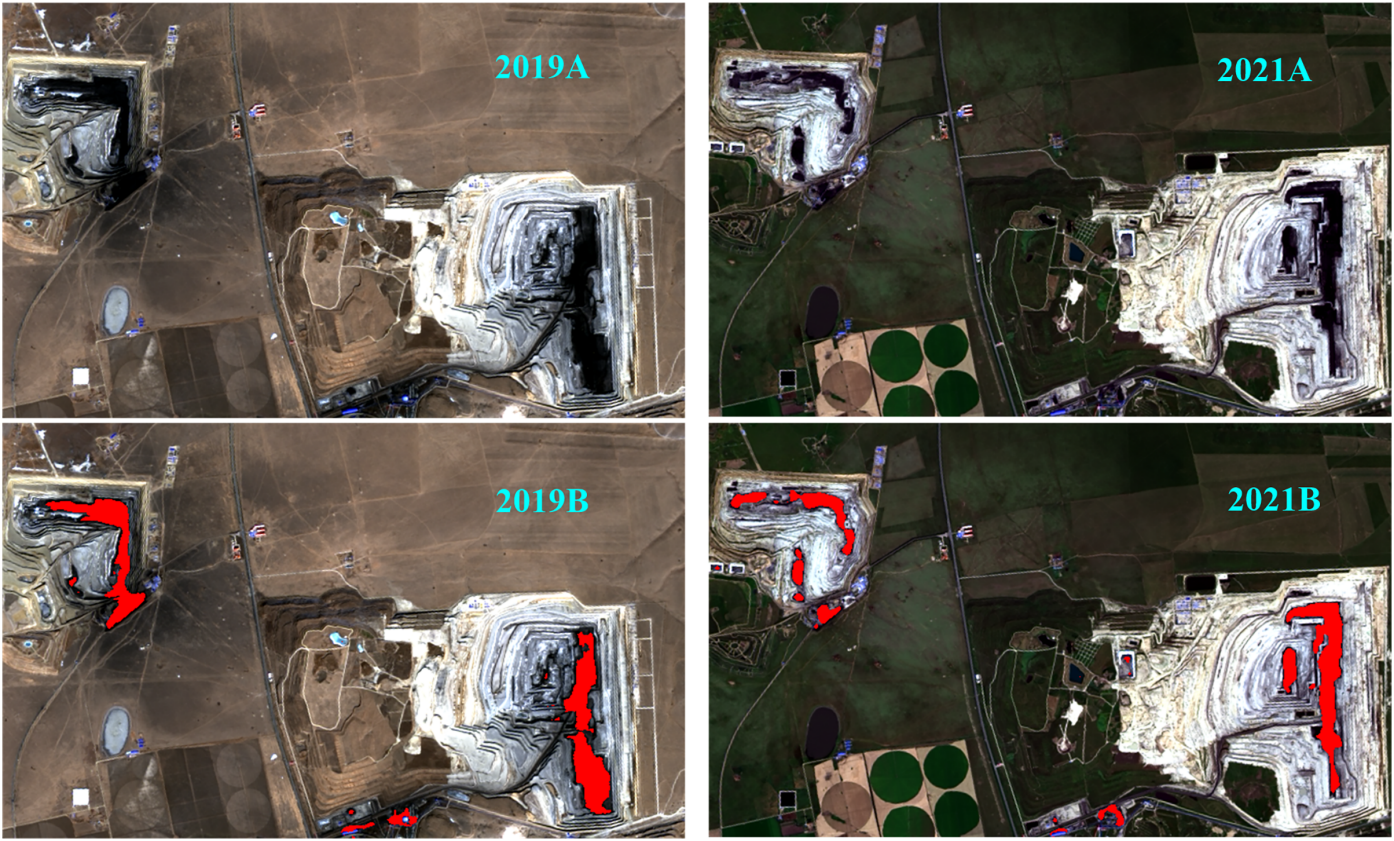
Semantic segmentation results of coal area.

The training samples are divided into coal and non-coal. We generated 6,000 samples as training set, 1,500 samples as validation set and 1,500 samples as test set by data augmentation method. We use MATLAB 2019b as a simulation tool under Windows 10 system. In the training process, we choose epochs to be 50, and the initial learning rate is 1e−3. After the model is trained, the classification accuracy of the test set is 99.65%. Applying the model to remote sensing images, Fig. 2 shows the semantic segmentation results of the classification model on remote sensing images. In Fig. 2, 2019A and 2021A are the original RGB remote sensing images; 2019A was taken on March 10, 2019, and 2021A was taken on August 22, 2021; 2019B and 2021B are semantic segmentation renderings, and the red part in these two figures represents the coal area. Through observation, we can see that the segmentation effect is good, almost all coal areas are extracted, and only a few buildings and coal gangue are marked as coal. Due to the small spectral difference between coal and coal gangue, there will be wrong identification areas. However, the model has good identification results for coal and coal gangue, which proves the effectiveness of the proposed method. We use deep network as a feature extractor and combine them with machine learning to make a classifier to further improve classification result. Deep network can effectively extract the characteristics of coal mine and help improve the classification accuracy of classifier. Therefore, the actual situation of opencast coal mines can be quickly understood through this model.

**Figure 3:**
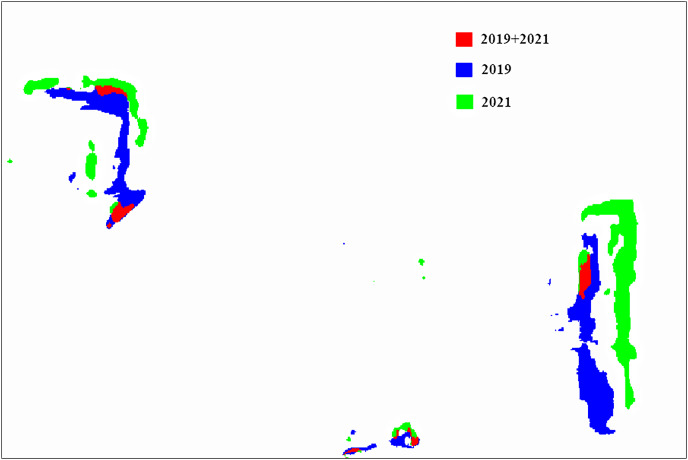
Coal area distribution in different years.

Figure 3 shows the distribution of coal areas in different years. The red area represents the overlap of coal areas in 2019 and 2021, the blue area represents 2019, and the green area represents 2021. We can see that the coal area changes greatly in different years, and the mining area has the trend of mining to the East. The distribution of coal area is basically consistent with the field investigation. This result can provide a rapid method for coal resource estimation, and lay a foundation for mine monitoring, environmental protection, *etc*.

### Regression performance

We use coefficient of determination (R) and root-mean-square error (RMSE) (Le et al., 2018a) to evaluate the performance of regression model, lower RMSE value and higher R value mean better model. Table 1 demonstrates the performance with different number of ensembles. The results are averaged over 10 experiments. Ten DELM models are randomly generated each time for selection. It can be seen from Table 1 that the larger the number of ensembles, the better the model performance. When the number of ensembles is 1, then the RMSE = 0.09, and R = 0.79. When the number of ensembles is 20, then the RMSE = 0.069, this value is reduced by 23%; the R = 0.88, this value is increased by 11%. The results show that the proposed method can improve the measurement performance of coal sulfur content. When the number of ensembles is from 1 to 5, the performance of the model improves rapidly, and after the number of ensembles is 10, the performance of the model does not improve significantly. As the number of DELMs increases, so does the training time. When the number of ensembles is 1, the training time is 0.16 s, and when the number of ensembles is 20, the training time is 2.84 s. The training time was increased by a factor of 17. Based on model performance and training time, we can conclude that 10 is a reasonable number of ensembles.

**Table 1:** Performance of the model with different numbers of DELMs.

Number of ensembles	RMSE	R	Training time (s)
1	0.090	0.79	0.16
2	0.088	0.80	0.31
5	0.081	0.84	0.73
7	0.080	0.84	0.97
10	0.073	0.87	1.41
15	0.071	0.87	2.11
20	0.069	0.88	2.84

**Figure 4:**
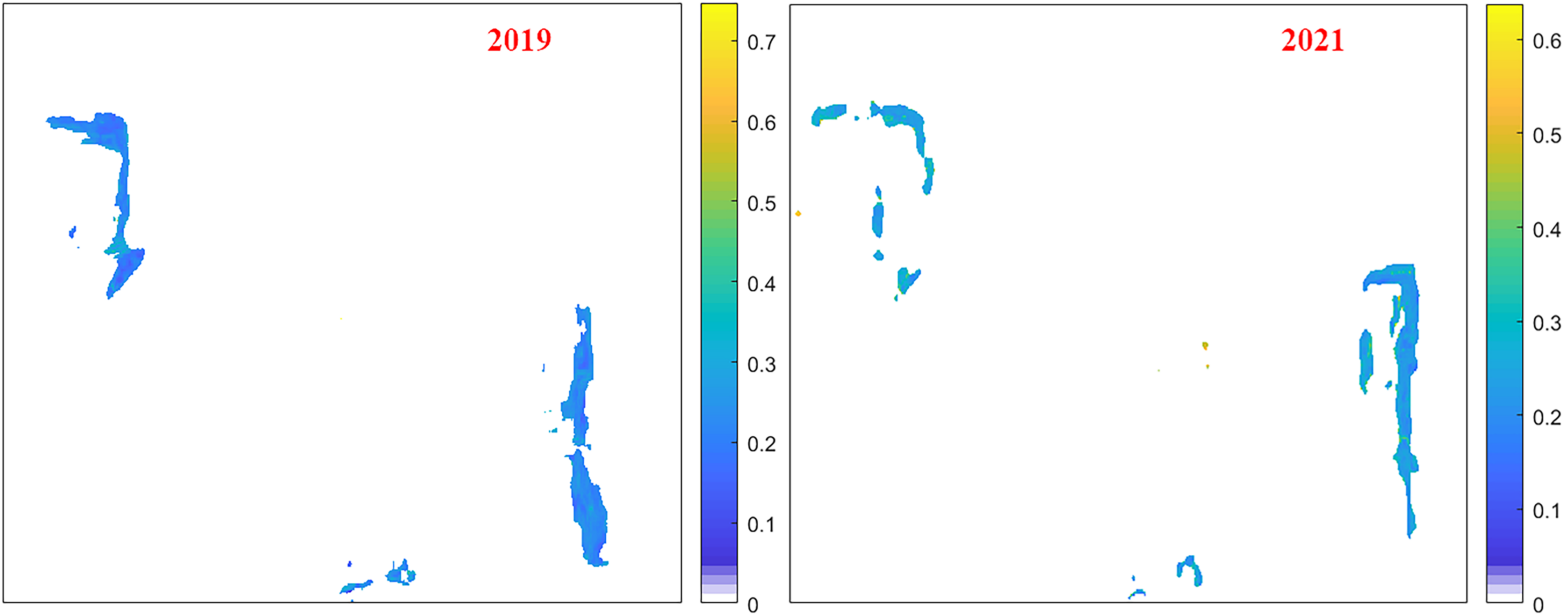
Mapping of sulfur content in the Baorixile coal mining area.

Figure 4 shows the effect of the proposed model for mapping sulfur content in the Baorixile coal mining area. We can see that the sulfur content in this mining area is relatively small, mainly in the range of 0.1–0.3%, indicating that the coal quality is good. Sulphur levels in 2021 are generally higher than in 2019. The results demonstrate the feasibility of the proposed method and provide a rapid method for evaluating sulfur content in field coal mining areas, and it provides a reliable basis for coal mining guidance and environmental protection policies.

### Comparison of different methods

This section compares several common coal analysis methods, including support vector machines (SVM) (Le et al., 2018a), partial least squares (PLS) (Liu et al., 2021). From Table 2, we can see that compared with other methods, the performance of PLS is worse; the performance of SVM and DELM methods are equal; the performance of our method is better than that of other methods. Specifically, the RMSE value of our method is 20% lower than that of SVM and DELM methods, and 25% lower than that of PLS. The R value of our method is much higher than other methods. The experimental results show that our method can effectively improve the prediction ability of coal sulfur content. This provides a fast and efficient method for coal content measurement.

**Table 2:** Comparison of different common methods.

Method	RMSE	R
SVM	0.092	0.78
PLS	0.098	0.75
DELM	0.090	0.79
Ours	0.073	0.87

Mao et al. (2014), proposed a coal extraction method by analyzing the reflectance spectral characteristics of coal. They built a coal classification model based on the difference between the 4th and 5th bands of Landsat, and obtained a classification accuracy of about 80%, which is much lower than our proposed method. Some physical and chemical analysis methods (Le et al., 2018a; Song et al., 2021; Liu et al., 2021) may exceed our proposed method in accuracy, but the time and cost of these methods are much higher than our method. Additionally, these methods can only assess one or a few locations in large coal mining areas. The method proposed by us can quickly, economically and large-scale map the distribution of sulfur content in coal mining areas, and the accuracy also meets the requirements of engineering. Overall, the proposed method can be applied to assess sulfur content and provide a reliable basis for real-time monitoring and production guidance in coal mining areas.

Although this study has achieved a series of results, it is difficult to further improve the accuracy of the regression model due to the limitation of satellite image resolution. On the other hand, the deep learning and machine learning parts of our model are carried out separately. Therefore, there are still many problems to be explored in future work.

## Conclusion

This study proposes a method to rapidly map sulfur content in coal mining areas. The method combines field and remote sensing data and builds an analytical model based on deep neural networks. The results show that the proposed method outperforms typical machine learning methods and some physical and chemical analysis methods. The RMSE value of our method is 20% lower than that of SVM and DELM methods, and 25% lower than that of PLS. Based on the results obtained, the proposed method can be applied to assess sulfur content and provide a reliable basis for real-time monitoring and production guidance in coal mining areas.

## Supplemental Information

10.7717/peerj-cs.2458/supp-1 Supplemental Information 1 Coal remote sensing and field data.

## Additional Information and Declarations

### Data Availability

The following information was supplied regarding data availability: The code is available at Zenodo: lebatuan. (2024). lebatuan/coalCNN: v1.0.0 (v1.0). Zenodo. https://doi.org/10.5281/zenodo.13748723.

## References

[ref-1] Albarakati HM, Khan MA, Hamza A, Khan F, Kraiem N, Jamel L, Almuqren L, Alroobaea R (2024). A novel deep learning architecture for agriculture land cover and land use classification from remote sensing images based on network-level fusion of self-attention architecture. IEEE Journal of Selected Topics in Applied Earth Observations and Remote Sensing.

[ref-2] Ali N, Fu X, Ashraf U, Chen J, Thanh HV, Anees A, Riaz MS, Fida M, Hussain MA, Hussain S, Hussain W, Ahmed A (2022). Remote sensing for surface coal mining and reclamation monitoring in the Central Salt Range, Punjab, Pakistan. Sustainability.

[ref-3] Bai J, Li Y, Li J, Yang X, Jiang Y, Xia ST (2022). Multinomial random forest. Pattern Recognition.

[ref-4] Begum N, Maiti A, Chakravarty D, Das BS (2021). Diffuse reflectance spectroscopy based rapid coal rank estimation: a machine learning enabled framework. Spectrochimica Acta Part A: Molecular and Biomolecular Spectroscopy.

[ref-5] Biswal SS, Raval S, Gorai AK (2019). Delineation and mapping of coal mine fire using remote sensing data–a review. International Journal of Remote Sensing.

[ref-6] Cai S, Zhang S, Wei Y, Sher F, Wen L, Xu J, Dang J, Hu L (2021). A novel method for removing organic sulfur from high-sulfur coal: migration of organic sulfur during microwave treatment with NaOH-H2O2. Fuel.

[ref-7] Cao J, Lin Z, Huang GB, Liu N (2012). Voting based extreme learning machine. Information Sciences.

[ref-8] Chen C, Tang Y, Guo X (2022). Comparison of structural characteristics of high-organic-sulfur and low-organic-sulfur coal of various ranks based on FTIR and Raman spectroscopy. Fuel.

[ref-9] Cheng G, Xie X, Han J, Guo L, Xia GS (2020). Remote sensing image scene classification meets deep learning: challenges, methods, benchmarks, and opportunities. IEEE Journal of Selected Topics in Applied Earth Observations and Remote Sensing.

[ref-10] Elhaki O, Shojaei K (2022). Output-feedback robust saturated actor–critic multi-layer neural network controller for multi-body electrically driven tractors with n-trailer guaranteeing prescribed output constraints. Robotics and Autonomous Systems.

[ref-11] He D, Le BT, Xiao D, Mao Y, Shan F, Ha TTL (2019). Coal mine area monitoring method by machine learning and multispectral remote sensing images. Infrared Physics & Technology.

[ref-12] Hou Z, Wang Z, Li L, Yu X, Li T, Yao H, Yan G, Ye Q, Liu Z, Zheng H (2022). Fast measurement of coking properties of coal using laser induced breakdown spectroscopy. Spectrochimica Acta Part B: Atomic Spectroscopy.

[ref-13] Le BT, Xiao D, Mao Y, He D (2018a). Coal analysis based on visible-infrared spectroscopy and a deep neural network. Infrared Physics & Technology.

[ref-14] Le BT, Xiao D, Mao Y, He D, Xu J, Song L (2019). Coal quality exploration technology based on an incremental multilayer extreme learning machine and remote sensing images. IEEE Transactions on Geoscience and Remote Sensing.

[ref-15] Le BT, Xiao D, Mao Y, He D, Zhang S, Sun X, Liu X (2018b). Coal exploration based on a multilayer extreme learning machine and satellite images. IEEE Access.

[ref-16] Liu K, He C, Zhu C, Chen J, Zhan K, Li X (2021). A review of laser-induced breakdown spectroscopy for coal analysis. TrAC Trends in Analytical Chemistry.

[ref-17] Luther A, Kostinek J, Kleinschek R, Defratyka S, Stanisavljević M, Forstmaier A, Dandocsi A, Scheidweiler L, Dubravica D, Wildmann N, Hase F, Frey MM, Chen J, Dietrich F, Nęcki J, Swolkień J, Knote C, Vardag SN, Roiger A, Butz A (2022). Observational constraints on methane emissions from Polish coal mines using a ground-based remote sensing network. Atmospheric Chemistry and Physics.

[ref-18] Ma Q, Wu J, He C, Fang X (2021). The speed, scale, and environmental and economic impacts of surface coal mining in the Mongolian Plateau. Resources, Conservation and Recycling.

[ref-19] Madhuanand L, Sadavarte P, Visschedijk AJH, Denier Van Der Gon HAC, Aben I, Osei FB (2021). Deep convolutional neural networks for surface coal mines determination from sentinel-2 images. European Journal of Remote Sensing.

[ref-20] Mao Y, Ma B, Liu S, Wu L, Zhang X, Yu M (2014). Study and validation of a remote sensing model for coal extraction based on reflectance spectrum features. Canadian Journal of Remote Sensing.

[ref-21] Petrovic J, Savovic J, Rankovic D, Kuzmanovic M (2022). Quantitative analysis of coal by laser-induced breakdown spectroscopy using tea co2 laser as the excitation source. Plasma Chemistry and Plasma Processing.

[ref-22] Qu BY, Lang BF, Liang JJ, Qin AK, Crisalle OD (2016). Two-hidden-layer extreme learning machine for regression and classification. Neurocomputing.

[ref-23] Sarıhan G, Kizgut S, Yilmaz S, Bilen M (2021). A new approach for the prediction of combustible sulphur in coal in terms of coal washability data and calorific value. International Journal of Oil, Gas and Coal Technology.

[ref-24] Sheta S, Afgan MS, Hou Z, Yao SC, Zhang L, Li Z, Wang Z (2019). Coal analysis by laser-induced breakdown spectroscopy: a tutorial review. Journal of Analytical Atomic Spectrometry.

[ref-25] Song W, Hou Z, Gu W, Wang H, Cui J, Zhou Z, Yan G, Ye Q, Li Z, Wang Z (2021). Industrial at-line analysis of coal properties using laser-induced breakdown spectroscopy combined with machine learning. Fuel.

[ref-26] Song L, Yu Y, Yan Z, Xiao D, Sun Y, Zhang X, Li X, Cheng B, Gao H, Bai D (2022). Rapid analysis of composition of coal gangue based on deep learning and thermal infrared spectroscopy. Sustainability.

[ref-27] Su H, Tang Z, Qiu J, Wang A, Yan XH (2024). Estimating the mixed layer depth of the global ocean by combining multisource remote sensing and spatiotemporal deep learning. International Journal of Digital Earth.

[ref-28] Tan K, Qiao J (2020). Development history and prospect of remote sensing technology in coal geology of China. International Journal of Coal Science & Technology.

[ref-29] Tang R, Liu Q, Zhong W, Lian G, Yu H (2020). Experimental study of SO2 emission and sulfur conversion characteristics of pressurized oxy-fuel co-combustion of coal and biomass. Energy & Fuels.

[ref-30] Thiruchittampalam S, Banerjee BP, Glenn NF, Raval S (2024). Geotechnical characterisation of coal spoil piles using high-resolution optical and multispectral data: a machine learning approach. Engineering Geology.

[ref-31] Xiao D, Le TTG, Doan TT, Le BT (2022). Coal identification based on a deep network and reflectance spectroscopy. Spectrochimica Acta Part A: Molecular and Biomolecular Spectroscopy.

[ref-32] Xiao D, Vu QH, Le BT, Ha TTL (2023). A method for mapping and monitoring of iron ore stopes based on hyperspectral remote sensing-ground data and a 3D deep neural network. Neural Computing and Applications.

[ref-33] Yan C, Qi J, Ma J, Tang H, Zhang T, Li H (2017). Determination of carbon and sulfur content in coal by laser induced breakdown spectroscopy combined with kernel-based extreme learning machine. Chemometrics and Intelligent Laboratory Systems.

[ref-34] Zeng X, Liu Z, He C, Ma Q, Wu J (2017). Detecting surface coal mining areas from remote sensing imagery: an approach based on object-oriented decision trees. Journal of Applied Remote Sensing.

